# Loss of *ctnnd2b* affects neuronal differentiation and behavior in zebrafish

**DOI:** 10.3389/fnins.2023.1205653

**Published:** 2023-07-03

**Authors:** Raquel Vaz, Steven Edwards, Alfredo Dueñas-Rey, Wolfgang Hofmeister, Anna Lindstrand

**Affiliations:** ^1^Department of Molecular Medicine and Surgery and Centre of Molecular Medicine, Karolinska Institutet, Stockholm, Sweden; ^2^Department of Applied Physics and Science for Life Laboratory, KTH Royal Institute of Technology, Stockholm, Sweden; ^3^Department of Clinical Genetics, Karolinska University Hospital, Stockholm, Sweden

**Keywords:** CTNND2, CRISPR/Cas9, zebrafish, neuronal development, swimming behavior

## Abstract

Delta-catenin (CTNND2) is an adhesive junction associated protein belonging to the family of p120 catenins. The human gene is located on the short arm of chromosome 5, the region deleted in Cri-du-chat syndrome (OMIM #123450). Heterozygous loss of *CTNND2* has been linked to a wide spectrum of neurodevelopmental disorders such as autism, schizophrenia, and intellectual disability. Here we studied how heterozygous loss of *ctnnd2b* affects zebrafish embryonic development, and larvae and adult behavior. First, we observed a disorganization of neuronal subtypes in the developing forebrain, namely the presence of ectopic *isl1-*expressing cells and a local reduction of GABA-positive neurons in the optic recess region. Next, using time-lapse analysis, we found that the disorganized distribution of *is1l-*expressing forebrain neurons resulted from an increased specification of Isl1:GFP neurons. Finally, we studied the swimming patterns of both larval and adult heterozygous zebrafish and observed an increased activity compared to wildtype animals. Overall, this data suggests a role for *ctnnd2b* in the differentiation cascade of neuronal subtypes in specific regions of the vertebrate brain, with repercussions in the animal’s behavior.

## 1. Introduction

CTNND2, also known as delta-catenin or neural plakophilin-related armadillo protein (NPRAP), is the only member of the catenin family of proteins expressed exclusively in the nervous system (Ho et al., 2000). Genetic changes affecting this gene have been shown to result in various neurodevelopmental disorders. The most known is Cri-du-chat syndrome (OMIM #123450), resulting from a large deletion of chromosome 5p with the hallmark features including an unusual high-pitched cry at birth, facial dysmorphology, poor growth, and severe intellectual disability. Interestingly, individuals in whom *CTNND2* was included in the deleted region showed more severe neurological symptoms (Medina et al., 2000; Mainardi et al., 2001; Sardina et al., 2014). Genetic changes affecting *CTNND2*, both structural variants and single nucleotide variants, have been associated with autism-spectrum disorder (Girirajan et al., 2013; Turner et al., 2015; Miller et al., 2020; Tuncay et al., 2022), intellectual disability with or without dyslexia-like learning difficulties (Belcaro et al., 2015; Hofmeister et al., 2015), neurodevelopmental delay (Asadollahi et al., 2014), attention deficit hyperactivity disorder or ADHD (Adegbola et al., 2020), depression (Kang et al., 2021), cerebral palsy (McMichael et al., 2014), anxiety (Nivard et al., 2014), epilepsy (van Rootselaar et al., 2017), and schizophrenia (Vrijenhoek et al., 2008). Even though it is not entirely understood how heterozygous loss or expression of *CTNND2* variants result in such disorders, research done in *in vitro* systems, such as neurons in culture, or animal models, have provided with some understanding of the possible role of CTNND2 in neurogenesis. In brief, CTNND2 plays a role in neuronal differentiation (Jones et al., 2002; Lee et al., 2016) and dendrite and synapse formation and maintenance (Israely et al., 2004; Kim et al., 2008; Arikkath et al., 2009; Matter et al., 2009; Turner et al., 2015; Gilbert and Man, 2016; van Rootselaar et al., 2017; Baumert et al., 2020; Ligon et al., 2020). These are, at least in part, regulated by CTNND2 via its interaction with the actin cytoskeleton, the ubiquitin proteasome pathway, and autophagy (Bareiss et al., 2010; Lee et al., 2016; Ligon et al., 2020). Expression studies in mice showed that *CTNND2* expression is high in neuronal precursors, downregulated during neuronal migration, and then increases again in post-mitotic neurons (Ho et al., 2000), supporting a dynamic role for CTNND2 in neurogenesis.

CTNND2 interacts with proteins such as N-Cadherin (Arikkath et al., 2009; Yuan et al., 2015; Gilbert and Man, 2016), Presenilin (Kim et al., 2006), RhoGTPases (Abu-Elneel et al., 2008; Lu et al., 2016; Donta et al., 2022), and SHANK3 (Hassani Nia et al., 2020). Interestingly, *de novo* point mutations and deletions affecting *SHANK3* causes Phelan-McDermid syndrome (OMIM # 606232), a developmental disorder characterized by neonatal hypotonia, global developmental delay, severely delayed speech, and autistic behavior (Uchino and Waga, 2013; Ricciardello et al., 2021).

Studies in mice have shown that loss of CTNND2 results in decreased learning ability and autism-like behaviors such as reduced sociability and increased anxiety (Israely et al., 2004; Wang et al., 2021). Conversely, overexpression of *CTNND2* in mice resulted in increased sociability and reduced anxiety (Ryu et al., 2019). We have previously shown that morpholino knock down of *ctnnd2b* but not *ctnnd2a* results in misplaced neural precursors. Here, we have generated stable Crispr/Cas9 *ctnnd2a* and *ctnnd2b* mutants and show that heterozygous loss of *ctnnd2b* affects the brain patterning of zebrafish embryos and the swimming behavior of both larvae and adults.

## 2. Materials and methods

### 2.1. Zebrafish husbandry

Adult zebrafish were maintained on a 14 h day/ 10 h night cycle at the Karolinska Institutet zebrafish core facility, KM-C. Embryos were produced by light-induced spawning, collected, and maintained in an incubator at 28.5°C until used in experiments. *Tg(isl1:GFP)rw0* transgenic (*isl:GFP* for short) and TL strains were used. All experiments were performed according to standard procedures and with permission from the Stockholm Ethical Board for Animal Experiments (ethics permit numbers 13063-2017, 14049-2019, and 14469-2022).

### 2.2. CRISPR/Cas9-mediated knockout of *ctnnd2* orthologues

Zebrafish mutant lines were established using a protocol adapted from Varshney et al. Varshney et al. (2015). sgRNAs and Cas9 protein mix was injected into 1-cell embryos and efficacy was tested using a PCR-STAT method (Carrington et al., 2015). The target sequence of the sgRNAs were in exon 6 of *ctnnd2a* 5’ GGTTCAGCGAGCGATACACC 3′ and exon 5 of *ctnnd2b* 5’ GGTGGAAGCGCCCGGGCCAG 3′. Primers used for genotyping were *ctnnd2a-F* 5’ TCACTCTCCTTACAGGTGACA 3′, *ctnnd2a-R* 5’ GGTTGCGTAGCCAGGTGTAT 3′, *ctnnd2b-F* 5’ CCAGGTCCTGAACTTTCTGC 3′, and *ctnnd2b-R* 5’ GGTCCTTCTGGATGCTGTCC 3′. Embryos were raised and founders identified by PCR and Sanger sequencing.

The stable *ctnnd2* mutant lines presented with short indels in the coding sequence resulting in a frameshift and premature termination of the protein sequence: *ctnnd2a* c.812_819del, p.S270TfsX61 and *ctnnd2b* c.1363_1369del, p.T456RfsX38 (Supplementary Figures S1A, S2). Non-sense mediated decay of mutant allele was evaluated using primers flanking the indel in *ctnnd2b* 5’ GACATTCCCTCGCGCTGGTTAC 3′ and 5’ CCCTCCAGCCAAATTCCCTGGG 3′ (forward and reverse primers, respectively). Amplification of *β-actin* was used as the reference gene with the primers 5’ CCGTGACATCAAGGAGAAGCT 3′ and 5’ TCGTGGATACCGCAAGATTCC 3′ (Supplementary Figure S1B).

Founders were outcrossed to wildtype or Tg (*isl:GFP)rw0* zebrafish and raised to adulthood. Given the lack of any severe phenotype, homozygous mutants were maintained as adults and outcrossed to generate heterozygous embryos used in the experiments.

### 2.3. Immunofluorescence and *In situ* hybridization

Embryos were fixed in 4% paraformaldehyde (PFA, HistoLabs) overnight at 4°C. Whole larvae or dissected brains were processed for immunofluorescence using standard protocol (Devine and Key, 2003). Primary antibody used was rabbit anti-GABA (Sigma, Cat# A2052, RRID: AB_477652) and secondary antibodies used were goat anti-rabbit Alexa 594 (Molecular Probes, Cat# A11012, RRID: AB_141359) and donkey anti-goat Alexa 546 (Molecular Probes, Cat#A11056, RRID: AB_142628). *In situ* staining was performed as previously described (Thisse and Thisse, 2008). The *nkx2.1* riboprobe was synthesized using the DIG labeling kit (Roche) using the following PCR template with a T7 RNA polymerase site attached to the 5′ end of the reverse primer (forward primer: 5’ TTGCCTCGTACAGACAACCC 3′, reverse primer: 5’ TAATACGACTCACTATAGGGGTCACCACGTCCTGCCATAA 3′). Embryos were mounted in gelvatol for imaging.

### 2.4. Imaging and data analysis

For confocal imaging, transgenic embryos fixed in 4% PFA or immunolabeled embryos were mounted rostrally or laterally in gelvatol mounting medium and imaged using a Zeiss LSM 700,800 or Olympus FV1000. Images where blinded and *isl:GFP* or GABA stained cells counted throughout individual confocal sections. Cell numbers were subsequently corelated back to the genotype of the embryos as identified by Sanger sequencing. Quantifications from multiple experiments/crosses were pooled together. For GABA-positive cell quantifications, data was obtained from two separate crosses of double heterozygous adults and one cross of double homozygous adults.

For light sheet imaging, 31hpf embryos were anesthetized in tricaine (MS222) and mounted in 1% low melting point agarose containing 1:2000 F-Z 050 fluorescent microspheres (Merck) in a glass capillary. The agarose cylinder was extruded into a sample chamber containing E3 medium at 28.5°C. Two-channel images were acquired using a Light Sheet Z.1(Carl Zeiss, Germany) with a water dipping 20X detection objective (W-Plan-APOCHROMAT-1.0NA) and dual side 10X illumination objectives (LSFM, 0.2NA). Samples were illuminated from two sides and Z-stacks were acquired from 3 angles (covering 90°) every 20 min for 16 h. Data from multiple angles was registered and deconvolved in the Multiview Reconstruction Fiji plugin (Preibisch et al., 2010). Briefly, beads were detected in one channel and used to register views and correct drift. Transformations were duplicated to the GFP channel and images were fused by multi-view deconvolution, performed on the GPU (Preibisch et al., 2014). Deconvolved images were rendered, and movies created in Imaris 9.0 (Bitplane).

### 2.5. Larval behavior analysis

Wildtype and *ctnnd2b^+/−^* embryos were produced and maintained in an incubator with light cycle (14 h light, 10 h dark) for 5 days, i.e., until the day of recording. The day before the experiment, embryos were placed in 48-well plates, one embryo per well, with a total of 1 mL of E3 medium per well. Recording was performed in a DanioVision Observation Chamber controlled by the EthoVision XT software (version 16, Noldus).

The visual motor response test was used to evaluate larvae behavior. Briefly, following a 5-min acclimatation in light, larvae were exposed to 5 iterations of 10 min light (100% light – equivalent to 3,000 LUX) and 10 min darkness (0% light). A video recording of larvae movements was acquired at a rate of 50 frames per second, resulting in evenly spaced frames every 20 milliseconds. Automatic single point detection was used to track individuals. After each recording, the video file was saved for offline inspection. A preliminary filtering using the distance per second was performed, with distances larger than 5 mm being discarded, as they may relate to ineffective larva detection. This was investigated by visualizing the trial video and understanding the cause of abnormal values. Data was analyzed using own scripts written in Python.

For thigmotaxis evaluation, each well was subdivided into an outer and inner zone. The inner zone was defined by a circle with 6.5 mm diameter at the center of the well (total diameter of the well is 10 mm). Total distance moved in the second dark iteration for each zone of the arena was calculated.

### 2.6. Adult behavior analysis

Five months old wildtype and *ctnnd2b^+/−^* zebrafish were used to assess response to a new environment, known as the novel tank test. Adult zebrafish were maintained according to standard procedures, in 3 L tanks at a maximum density of 20 fish per tank, until the time of the experiment. Recording was done between 9 am-12 pm in consecutive days and, following the recording, animals were placed in new tanks. A total of 17 animals per genotype were used in the test. For the recording, two tanks (17x11x19.5 cm LxWxH) were placed side by side and filled with water. The sides of the tanks were taped to avoid external visual stimulation. A camera was placed in front of the tanks to record the animals’ lateral and vertical movements. Recording started before the fish were transferred to the tanks and set to 16 min, to guarantee a full 15-min recording of every animal. For each recording, one fish from each genotype was used and were alternated between the recording tanks. Both genders were used. Tanks were rinsed and filled with fresh water between recordings. Tracking of the movements was performed using the Ethovision XT software and corrected when needed. For the analysis, the tank was subdivided into bottom and top zones (bottom corresponding to the bottom 1/3 of the tank and top corresponding to the rest of the tank).

### 2.7. Statistical analysis

Quantifications were statistically compared with GraphPad Prism version 9. For quantifications that fit the normal distribution of values, parametric tests were used. If a normal distribution of values was not observed, non-parametric tests were performed instead. Tests used are named in the results section. Statistical significance was considered when *p* < 0.05. Non-significant differences were not plotted in the graphs.

## 3. Results

### 3.1. Loss of *ctnnd2b* but not *ctnnd2a* results in misplaced neurons

**Figure 1:**
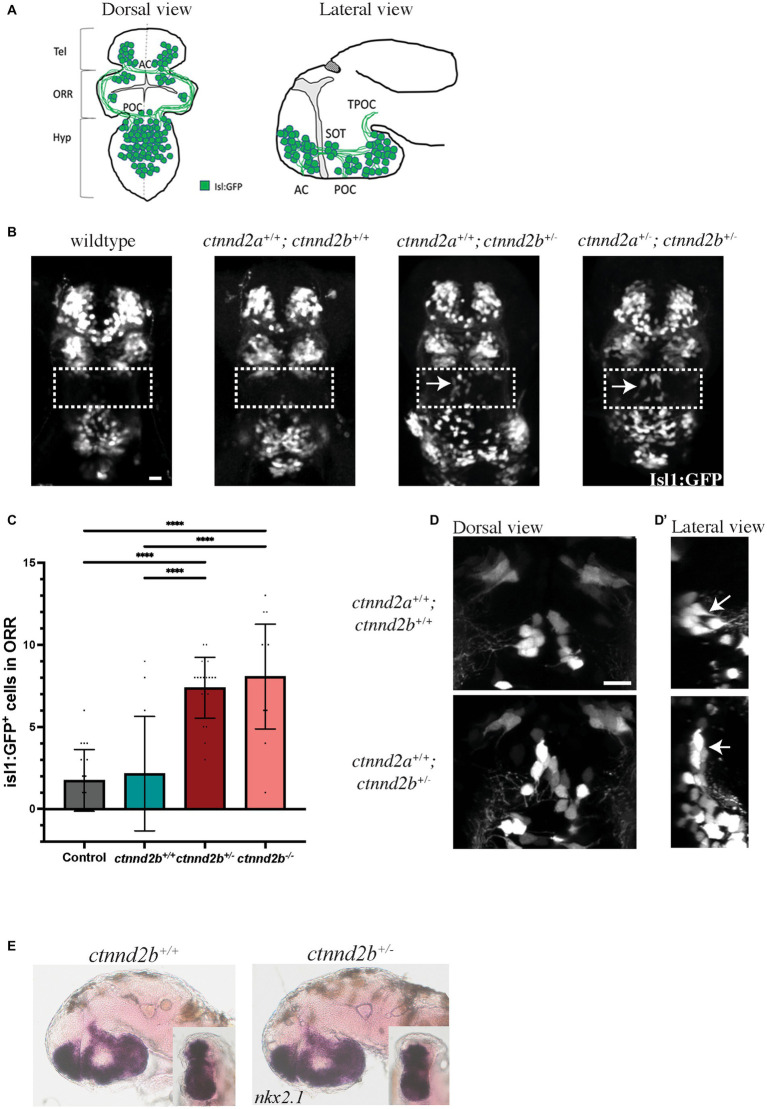
FIGURE 1 (Continued)**(A,B)** Schematic illustration of the *isl1:GFP*-expressing neuron population in the forebrain of wildtype 48 hpf zebrafish embryos. **(B)** Heterozygous loss of *ctnnd2b* results in ectopic *isl1:GFP*-expressing cells in the ORR (arrows), not detected in control embryos. Wildtype: embryos obtained from an incross of *Tg(isl1:GFP)* line; *ctnnd2a^+/+^,ctnnd2b^+/+^*: non-carriers from the incross of the *ctnnd2a^+/−^,ctnnd2b^+/−^* line. **(C)** Quantification of Isl1:GFP-positive neurons in the ORR confirmed that heterozygous and homozygous loss of *ctnnd2b* result in ectopic Isl1-positive cells in the region. **(D)** In *ctnnd2b^+/−^* embryos the ectopic Isl1:GFP-positive cells display a vertical rather than horizontal orientation (arrows, lateral view in D′) and the cluster seems more disorganized when compared to these cells in control (*ctnnd2a^+/+^,ctnnd2b^+/+^*) embryos. **(E)** Control and *ctnnd2b^+/−^* 54 hpf embryos show no difference in *nkx2.1* expression, investigated in lateral and rostral (inset) views. Dashed box: ORR; Tel: telencephalon; ORR: optic recess region; Hyp: hypothalamus; AC: anterior commissure; POC: post-optic commissure; SOT: supraoptic tract; TPOC: tract of the POC; ****p < 0.0001. Scale bar =10 μm.

The zebrafish genome contains two orthologs for the human *CTNND2*, *ctnnd2a* and *ctnnd2b*, most likely due to a genome-wide duplication event that occurred during *teleost* evolution (Glasauer and Neuhauss, 2014). We have previously shown that reduction of *ctnnd2b* expression but not *ctnnd2a* using antisense morpholinos in zebrafish embryos results in ectopic *isl1-*expressing neurons in the forebrain (Hofmeister et al., 2015). The ectopic Isl1:GFP-positive cells are found in the optic recess region (ORR), which can be identified as a distinct morphological entity in the brain of 48 h post fertilization (hpf) embryos (Figure 1A), located between the telencephalon (tel) and the hypothalamus (hyp), and flanked by the anterior and the postoptic commissure (AC and POC, respectively) (Affaticati et al., 2015).

To further investigate these findings, we created stable knockout lines for *ctnnd2* with CRISPR/Cas9. The changes generated by the genome editing resulted in frameshift mutations that introduced a premature stop codon to the coding sequence: *ctnnd2a* c.812_819del p.S270TfsX61, and *ctnnd2b* c.1363_1369del p.T456RfsX38 (Supplementary Figures S1, S2). For nomenclature simplicity, the mutant allele is represented by ‘-‘symbol, e.g., *ctnnd2b^−/−^* representing the homozygous *ctnnd2b* null genotype. Similarly to the knockdown of *ctnnd2* with morpholinos, heterozygous loss of *ctnnd2b*, but not *ctnnd2a*, resulted in ectopic *isl1*-expressing neurons in the ORR, seen in 54 hpf embryos (arrows, Figure 1B, Supplementary Figure S3A). Moreover, both heterozygous and homozygous *ctnnd2b* null embryos showed the same phenotype (Supplementary Figure S3A). Quantification of the number of Isl1:GFP-positive cells in the ORR showed a significant increase in both *ctnnd2b^+/−^* and *ctnnd2b^,−/−^* (*n* = 13–21 embryos per group, one-way ANOVA, ****p < 0.0001, Figure 1C). Interestingly, confocal analysis at higher magnification showed that the ectopic neurons display a vertical rather than horizontal orientation and the cluster seems disorganized compared to neurons in wildtype embryos (Figure 1D, lateral view D′, arrows). Ectopic neurons are still present at later stages, e.g., 6 days post fertilization (dpf) (arrows, Supplementary Figure S3B), suggesting the mispatterning of the brain may be maintained throughout development and into adulthood.

### 3.2. Heterozygous loss of *ctnnd2b* results in abnormal GABAergic neurogenesis

In mammals, ISL1 has been shown to form a complex with LHX3 to promote cholinergic gene expression in basal forebrain cholinergic neurons (Cho et al., 2014). This neuronal population is involved in attention, memory, reward pathways, and motor activity (Allaway and Machold, 2017). Forebrain cholinergic neurons have an embryonic origin in the ventral telencephalon, in a region that expresses the transcription factor *nkx2.1*. The *nkx2.1* expression domain in zebrafish where the *isl1*-expressing forebrain cells reside encompasses the ventral telencephalon, optic recess region, ventral diencephalon, and hypothalamus (Manoli and Driever, 2014). *In situ* analysis revealed no difference in patterning of the *nkx2.1* expression region between wildtype and heterozygous embryos (Figure 1E), suggesting normal patterning of this region at early developmental stages.

**Figure 2:**
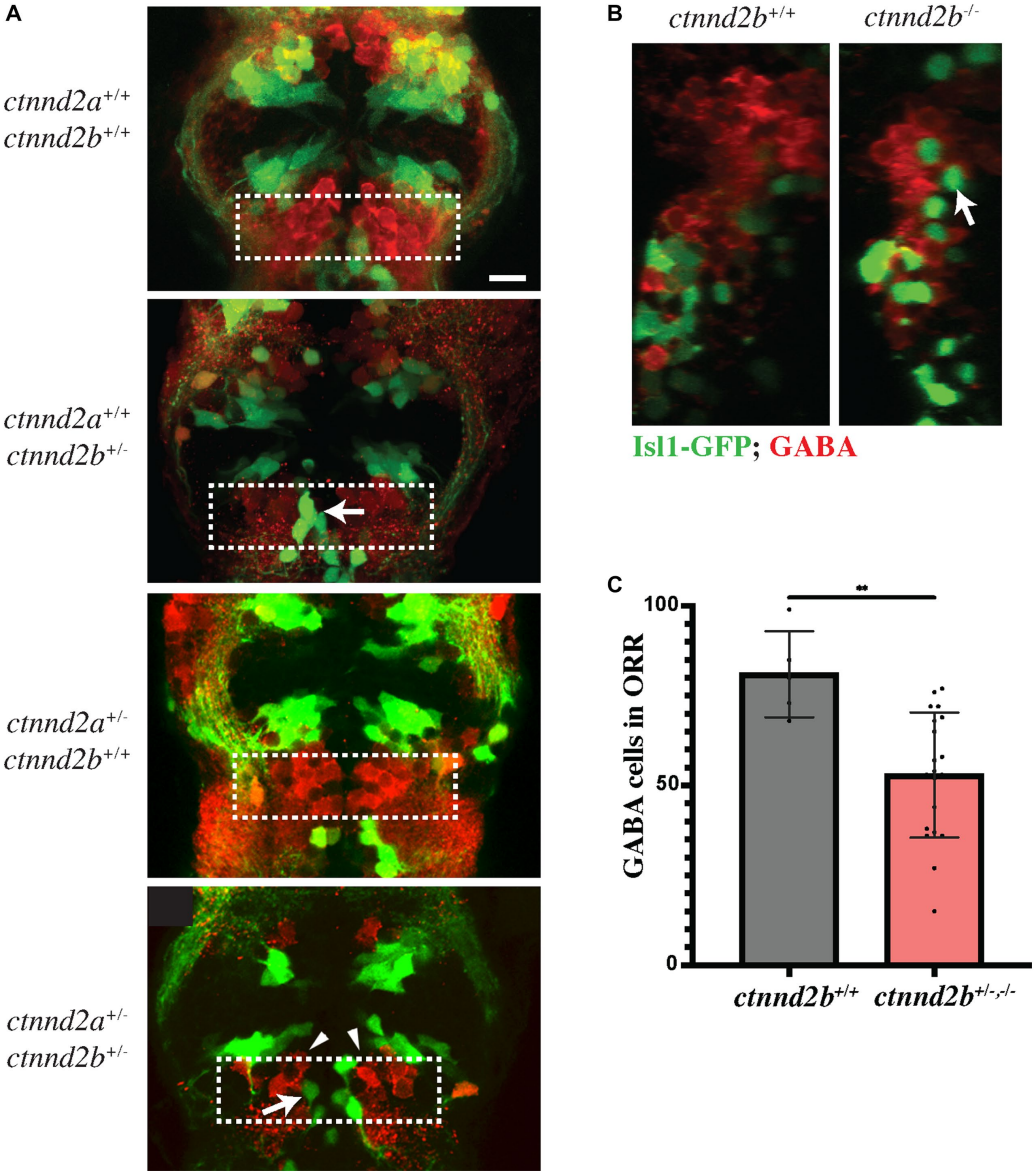
**(A)** Heterozygous loss of *ctnnd2b* affects GABA neuronal specification in the ORR, resulting in a significant decrease in the number and organization of GABA-positive cells in the ORR (dashed box, arrowheads in **A**; **C**). **(A,B)** Isl:GFP-positive cells are negative for GABA (arrows). **p = 0.0017. Scale bar =10 μm.

The ventral telencephalon is also the birthplace of GABAergic neurons. The choice as to whether *nkx2.1* expressing cells in the ventral telencephalon become GABAergic or cholinergic is due to a distinct expression of a combination of transcription factors. Neurons fated to become GABAergic express high levels of the transcription factor *lhx6* (Liodis et al., 2007). Conversely, in post-mitotic *nkx2.1* positive neurons expressing high levels of *isl1* and *lhx8,* the expression of *lhx6* is suppressed and these become forebrain cholinergic neurons (Elshatory and Gan, 2008; Cho et al., 2014). Thus, the differentiation into GABAergic neurons is inhibited by the expression of *isl1* (Fragkouli et al., 2009). We therefore decided to assess the expression of *GABA* in *ctnnd2b* mutant embryos. As expected, GABAergic neurons in diencephalon and ORR are intermingled but rarely overlapping with Isl1:GFP-positive cells (*ctnnd2a^+/+^,ctnnd2b^+/+^*), and ectopic Isl1-positive neurons also lack GABA expression (arrows, Figures 2A,B). Moreover, the population of GABA-positive neurons in the ORR appears to be more loosely packed together in *ctnnd2b^+/−^* embryos than in controls (arrowheads, Figure 2A). Interestingly, quantification of GABAergic neurons throughout the images taken at the ORR confirmed a reduction in GABA-positive neurons in heterozygous *ctnnd2b* embryos in addition to the cell disorganization (n = 5–20 embryos, Mann–Whitney test, **p = 0.0017; Figure 2C).

### 3.3. Increased *isl1*-expressing cells at ORR is due to abnormal neuronal specification

**Figure 3:**
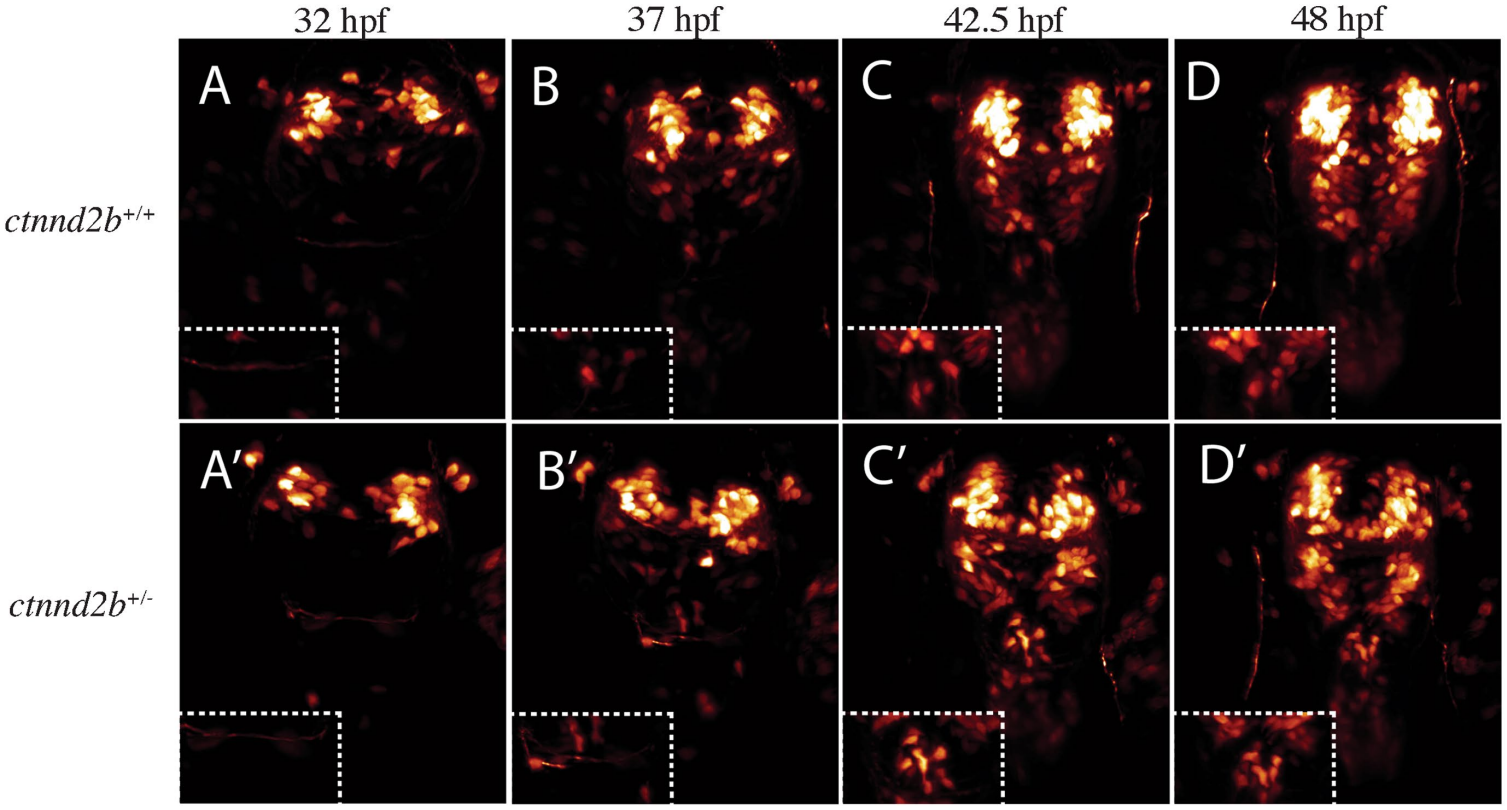
Increase in Isl1-positive cells is likely due to misspecification rather than migration defects. 3D renders of timelapse imaged *ctnnd2b*^+/+^ **(A-D)** and *ctnnd2b^+/−^* embryos **(A’-D’)** with highlighted ORR (insets). Embryos at 32 hpf, prior to appearance of neurons at ORR, are shown in **(A,A’)**. Isl1:GFP-positive neurons start appearing at the ORR by 37 hpf **(B,B’)**, and detected at 42.5 hpf **(C,C’)** and 48 hpf **(D,D’)**. Insets show the ORR at the corresponding timepoints. By the end of recording only a few *isl1:GFP*-expressing cells are found in *ctnnd2b*^+/+^ embryos compared to *ctnnd2b*^+/−^ embryos.

To address if the observed phenotype was a result of migration defects or abnormal specification, we imaged embryos between 32 and 48 hpf. Given the lack of phenotypes of embryos carrying the *ctnnd2a^+/−^* genotype, the following experiments were performed only on *ctnnd2b* wild type and mutant embryos (on a *ctnnd2a^+/+^* background). Most patients, including those we previously reported (Hofmeister et al., 2015) present with heterozygous loss of *CTNND2*. Since both *ctnnd2b^+/−^* and *ctnnd2b^−/−^* zebrafish embryos developed the same phenotype, we continued the experiments on *ctnnd2b^+/−^* embryos. Staging was additionally assessed by growth of visible axon tracts such as the trigeminal and post-optic commissure. Initially, the number of *isl1:GFP-*expressing cells were similar between transgenic control and heterozygous embryos (*ctnnd2b^+/−^*) (compare Figures 3A–A’, Supplementary Movie S1). However, as development proceeds, we see a progressive increase in the number of Isl1:GFP-positive cells arising in the ORR of *ctnnd2b^+/−^* embryos (compare Figures 3B,C–B’,C’, Supplementary Movie S1) that are not traceable to other regions of the embryo from earlier timepoints, while in the control there were only a very limited number of Isl1:GFP-positive cells detected (compare Figures 3D–D’, Supplementary Movie S1). The finding suggests that in the mutant embryos more cells from the ORR become committed to the Isl1-lineage compared to control embryos.

### 3.4. Reduction in Ctnnd2b affects swimming behavior

**Figure 4:**
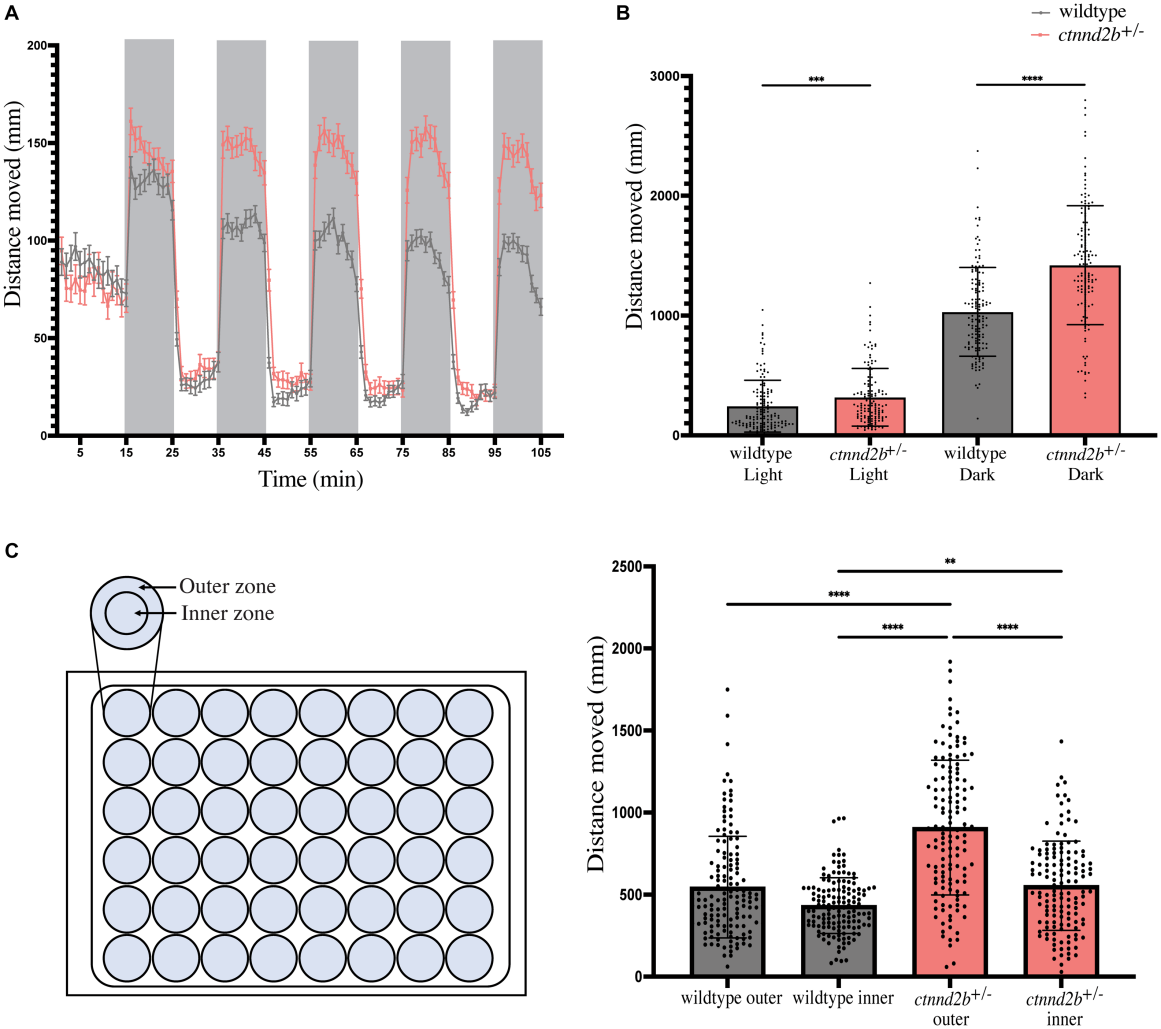
**(A)** Assessment of the swimming performance using the visual motor response test showed an increase in activity from *ctnnd2b^+/−^* 5 dpf larvae compared to wildtype larvae when exposed to darkness (shaded time windows). **(B)** Quantification of total distance moved per light condition showed that mutant larvae moved significantly move in both light and dark environments. **(C)** Evaluation of swimming patterns during a dark iteration of the test showed a statistically significant increase preference to swimming in the outer zone of the arena, or thigmotaxis, from the *ctnnd2b^+/−^* larvae compared to wildtype controls. **p = 0.0033, ***p = 0.0006, ****p < 0.0001.

To investigate if and how the changes in the brain patterning of *ctnnd2b^+/−^* embryos may affect their swimming behavior, we performed the visual motor response test on 5 dpf larvae (Vaz et al., 2019). Briefly, larvae were exposed to five consecutive cycles of light and dark and the distance traveled throughout the test was determined. Zebrafish larvae are known to be more active in the dark than in light conditions, which we also observed (Figure 4A). Interestingly, *ctnnd2b^+/−^* presented with a larger increase in swimming activity when exposed to darkness compared to wildtype larvae (Figures 4A,B). Quantification of the distance moved for each light condition (average of the total distance moved during each 10-min light condition) per fish was calculated. We found a statistically significant increase in the distance moved in both light and dark conditions by *ctnnd2b^+/−^* than wildtype larvae (n_wildtype_ = 138 and n*_ctnnd2b+/−_* = 133 embryos, Mann–Whitney test, ***p = 0.0006, ****p < 0.0001; Figure 4B). Another swimming behavior that can be evaluated is thigmotaxis, which is the preference for larvae to swim along the walls of the dish rather than in the center, that correlates with anxiety (Colwill and Creton, 2011; Schnorr et al., 2012). To assess if *ctnnd2b^+/−^* larvae presented with a different thigmotaxic behavior when compared to wildtype larvae, the arena was subdivided into an outer and inner zone (Figure 4C). The total distance traveled within each zone during one dark iteration of the visual motor response test was calculated. Mutant larvae presented with an overall increased activity in both zones compared to wildtype larvae (n_wildtype_ = 140 and n*_ctnnd2b+/−_* = 137 embryos, Kruskal-Wallis test, **p = 0.0033, ****p < 0.0001; Figure 4C). While wildtype embryos showed no difference between the distance moved in the outer and inner zones (*p* = 0.0663), the *ctnnd2b^+/−^* larvae moved significantly more in the outer zone compared to the inner zone of the arena (****p < 0.0001; Figure 4C), suggesting an increased anxiety-like behavior from the *ctnnd2b^+/−^* larvae.

**Figure 5:**
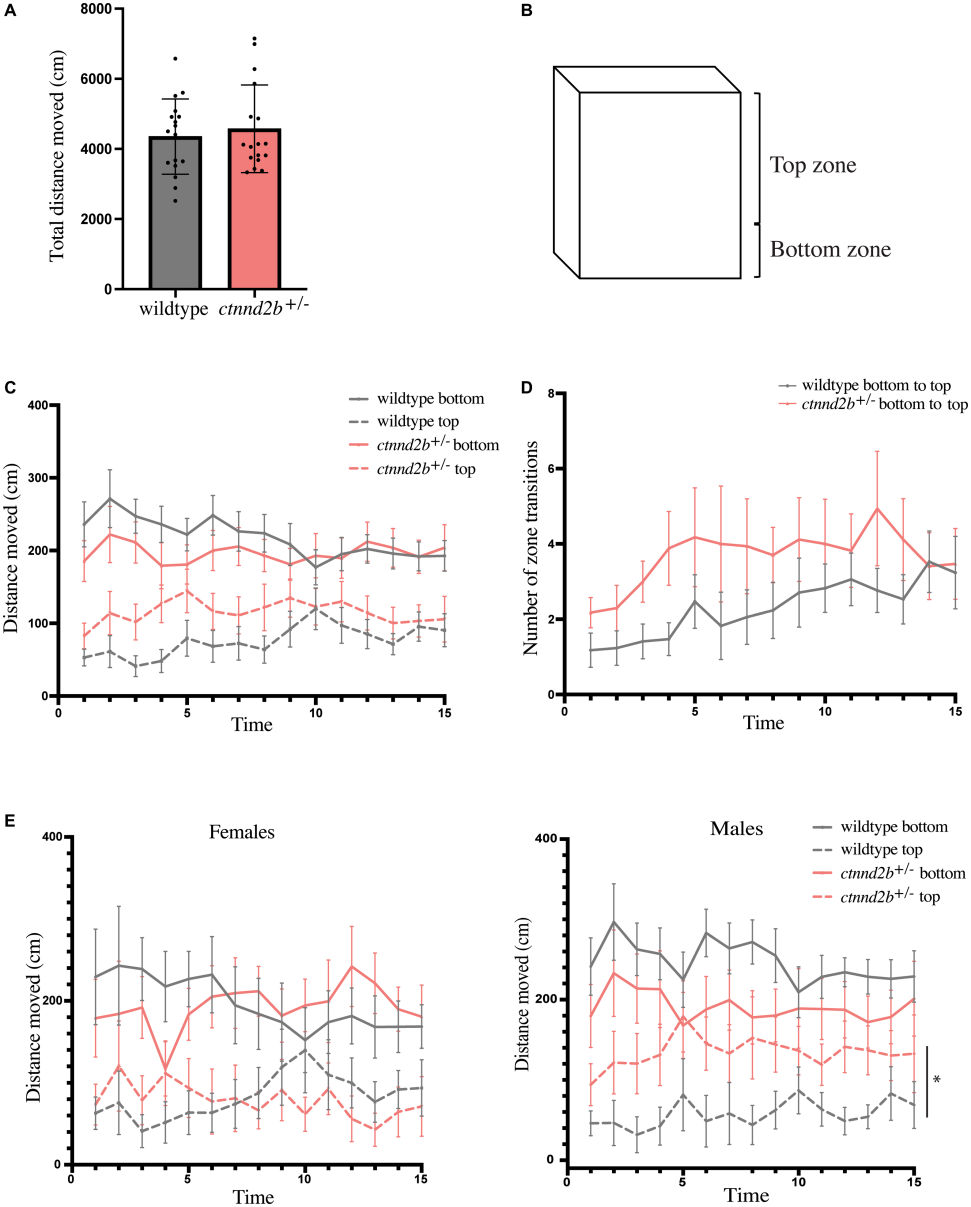
Behavior of adult individuals was evaluated using the novel tank test. **(A)** Total distance moved for the duration of the test was similar between *ctnnd2b^+/−^* and wildtype individuals. **(B)** Anxiety-like behavior was tested by defining a top and bottom zone of the tank used in the test. Quantification of the distance moved per minute per zone **(C)** and the number of bottom-to-top transitions during the test **(D)** showed no difference between genotypes. **(E)** While no difference in the distance moved per zone was found between females, heterozygous mutant males show a statistically significant increase in the distance moved in the top zone of the tank compared to wildtype individuals. Graphs: mean ± SEM, *p = 0.0252.

Next, we wanted to assess whether adult behavior was also affected. To do so, the novel tank test was performed, where one fish was transferred to a new tank and the swimming behavior was recorded. The total distance moved per fish for the 15 min of recording analyzed was similar between genotypes (n = 17 fish per genotype, t-test, *p* = 0.5829; Figure 5A). Even though there was no difference in the total distance swam by the animals, we investigated whether there was a preference for any specific zone in the tank. To address this question, the tank area was divided into bottom zone, the lower third of the tank height, and top zone, the upper area of the tank (Figure 5B). Frequency (time spent on each zone, not shown), distance, and number of transitions between the zones were calculated. We found a tendency for *ctnnd2b^+/−^* zebrafish to swim more in the top zone compared to wildtype animals (dashed lines, Figure 5C), however the variability between individuals with the same genotype is large, hence the differences are not statistically significant (two-way ANOVA, *p* = 0.4673 and *p* = 0.0761 for bottom and top zones; Figure 5C). Similar findings were observed when the number of transitions between the bottom and top zones were assessed (two-way ANOVA, p ≈ 0.16 for both bottom-to-top and top-to-bottom transitions; Figure 5D). It has been widely reported that male patients present with more severe changes in behavior than females. Therefore, we analyzed males and females separately. We found no significant difference between the distance moved throughout time between females from each genotype (two-way ANOVA, *p* = 0.9283 and *p* = 0.06961 for bottom and top zones, respectively) and in the bottom zone between males (two-way ANOVA, *p* = 0.2077), due to a large individual variability. However, mutant males move more in the top zone of the tank than wildtype males (two-way ANOVA, *p = 0.0252; Figure 5E), resembling a reduction in novelty-induced anxiety behaviors previously reported.

## 4. Discussion

To study how Ctnnd2 affects zebrafish development and behavior we generated and investigated a stable zebrafish knock out line. Using this approach, opposed to transient knock down of gene expression, we were able to phenotype older embryos, larvae, and adult individuals.

Our results show that, in developing embryos, *ctnnd2b* haploinsufficiency results in ectopic specification of Isl1-positive cells at the ORR. Since in this region of the brain cholinergic and GABAergic neurons are specified, it is plausible that an ectopic specification of Isl1-positive cells affect the neuronal pattering in the ORR. Previous publications have shown that Isl1, via its interaction with Lhx8 in the forebrain, promotes cholinergic specification (Elshatory and Gan, 2008; Cho et al., 2014). In fact, choline acetyltransferase is expressed in the region from early developmental stages (Rima et al., 2020; Ncube et al., 2022). We therefore suggest that the ectopic Isl1-positive cells may also differentiate into cholinergic neurons. In *ctnnd2b^+/−^* embryos we found a reduction of the number of GABA neurons in the ORR, suggesting that either directly or indirectly, ectopic Isl1 cells affect the local differentiation and organization of the GABA neurons in that region. Reduced GABAergic signaling has already been observed in individuals with autism (Fatemi et al., 2009; Oblak et al., 2011; Robertson et al., 2016), and genes encoding GABA receptor subunits have been associated with autism in both linkage and copy number studies (Ma et al., 2005). Mutations in *CNTNAP2*, an autism susceptibility gene, also results in a reduction in GABAergic neurons as well as hyperactivity, and changes in behavior in mice and zebrafish (Penagarikano et al., 2011; Hoffman et al., 2016). These findings strongly suggest that the imbalance or dysfunction of the GABAergic system is a candidate cause of autism, and that CTNND2 may have a role in the regulation of GABAergic neuron identity and the onset of this neurodevelopmental disorder.

Behavior assessment tests have been previously used in zebrafish to assess swimming patterns and test the influence of genes, toxicants, and other compounds (Egan et al., 2009; Wong et al., 2010; Ali et al., 2012; de Esch et al., 2012; Lange et al., 2012; Vignet et al., 2013; Ziv et al., 2013; Kim et al., 2014, 2017; Spulber et al., 2014; Crosby et al., 2015; Li et al., 2015). Here, we compared *ctnnd2b^+/−^* larvae and adults to wildtype individuals. At both developmental stages we found an increase in swimming activity and exploratory behavior. It has been reported previously that healthy zebrafish larvae move more when in the dark than light (MacPhail et al., 2009; Padilla et al., 2011; de Esch et al., 2012; Li et al., 2015). Interestingly, we observe that larvae missing one copy of *ctnnd2b* present with a more pronounced increase in activity when exposed to darkness than controls, resembling the changes reported in the Cntnap2 zebrafish autism model mentioned above (Hoffman et al., 2016), as well as in a model for *lphn3.*1-related ADHD (Lange et al., 2012). The changes in thigmotaxis we observe in *ctnnd2b^+/−^* larvae are also consistent with changes seen in other zebrafish models (Lockwood et al., 2004; Schnorr et al., 2012). Conversely, some studies presenting zebrafish models for Rett syndrome and autism have shown an opposite effect on behavior, namely in the distance moved in the visual motor response test and thigmotaxis (Pietri et al., 2013; Liu et al., 2018). These opposite effects are interesting but remain to be understood.

In adult *ctnnd2b^+/−^* we found an increase in the distance swam in the tank top zone compared to wildtype controls. These differences were more pronounced during the first 10 minutes of the test and were only significantly different between male zebrafish (Figures 5C,D). Other studies have shown similar findings, that the exploration to the top zone increases over time (Cachat et al., 2011; Vignet et al., 2013). Previous studies have shown that exposure to compounds that cause autism-like changes in behavior result in increased activity and exploratory behavior, resembling the *ctnnd2b^+/−^* fish’s behavior observed in our study (Maaswinkel et al., 2013). One limitation of our setup was the use of a single camera recording, instead of two cameras (from the side and the top of the tank) that would allow us to better triangulate the movements of the animals and assess other behavior features such as freezing and turn patterns. Nevertheless, supporting our observations, zebrafish models for fragile X syndrome and Down syndrome also presented with an increased vertical activity, with knockout animals spending more time in the top zone of the tank than controls (Ng et al., 2013; Kim et al., 2014, 2017). These findings suggest that there may be an imbalance between the excitatory and inhibitory neuronal signals or that the mutant animals present with a lack of impulsivity control, which we are not able to decipher yet.

Our study supports a role for CTNND2 haploinsufficiency in the etiology of neurodevelopmental disorders due to abnormal GABA neuronal differentiation in the forebrain. This haploinsufficiency affects the behavior of the carriers in similar ways to other neurodevelopmental disorders. Knowing the affected pathway causing the onset of the disorder offers the opportunity to test compounds able to rescue if not all, some phenotypes. Several compounds have efficaciously rescued abnormal behaviors in mutant animals in disorders such as ADHD (Lange et al., 2012) and ASD (Hoffman et al., 2016). Estrogens, for example, were successfully able to rescue the behavior changes in *cntnap2ab*-related ASD (Hoffman et al., 2016), suggesting that they could also be efficacious in our disease model.

## Data availability statement

The raw data supporting the conclusions of this article will be made available by the authors, without undue reservation.

## Ethics statement

The animal study was reviewed and approved by The Stockholm Ethical Board for Animal Experiments.

## Author contributions

RV, WH, and AL conceptualized the work. RV, SE, AD-R, and WH performed the experiments and analyzed and interpreted the data. RV drafted the manuscript. All authors contributed to the article and approved the submitted version.

## Funding

This study was funded by the Brain Foundation (Hjärnfonden, FO2022-0256), the Swedish research council (2019–02078) and Region Stockholm.

## Conflict of interest

The authors declare that the research was conducted in the absence of any commercial or financial relationships that could be construed as a potential conflict of interest.

## Publisher’s note

All claims expressed in this article are solely those of the authors and do not necessarily represent those of their affiliated organizations, or those of the publisher, the editors and the reviewers. Any product that may be evaluated in this article, or claim that may be made by its manufacturer, is not guaranteed or endorsed by the publisher.
