# Supplementary figures and images for: Loss of ctnnd2b affects neuronal differentiation and behavior in zebrafish
Source: Front Neurosci. 2023 Jul 3;17:1205653. doi: 10.3389/fnins.2023.1205653 (PMC10351287; doi:10.3389/fnins.2023.1205653)

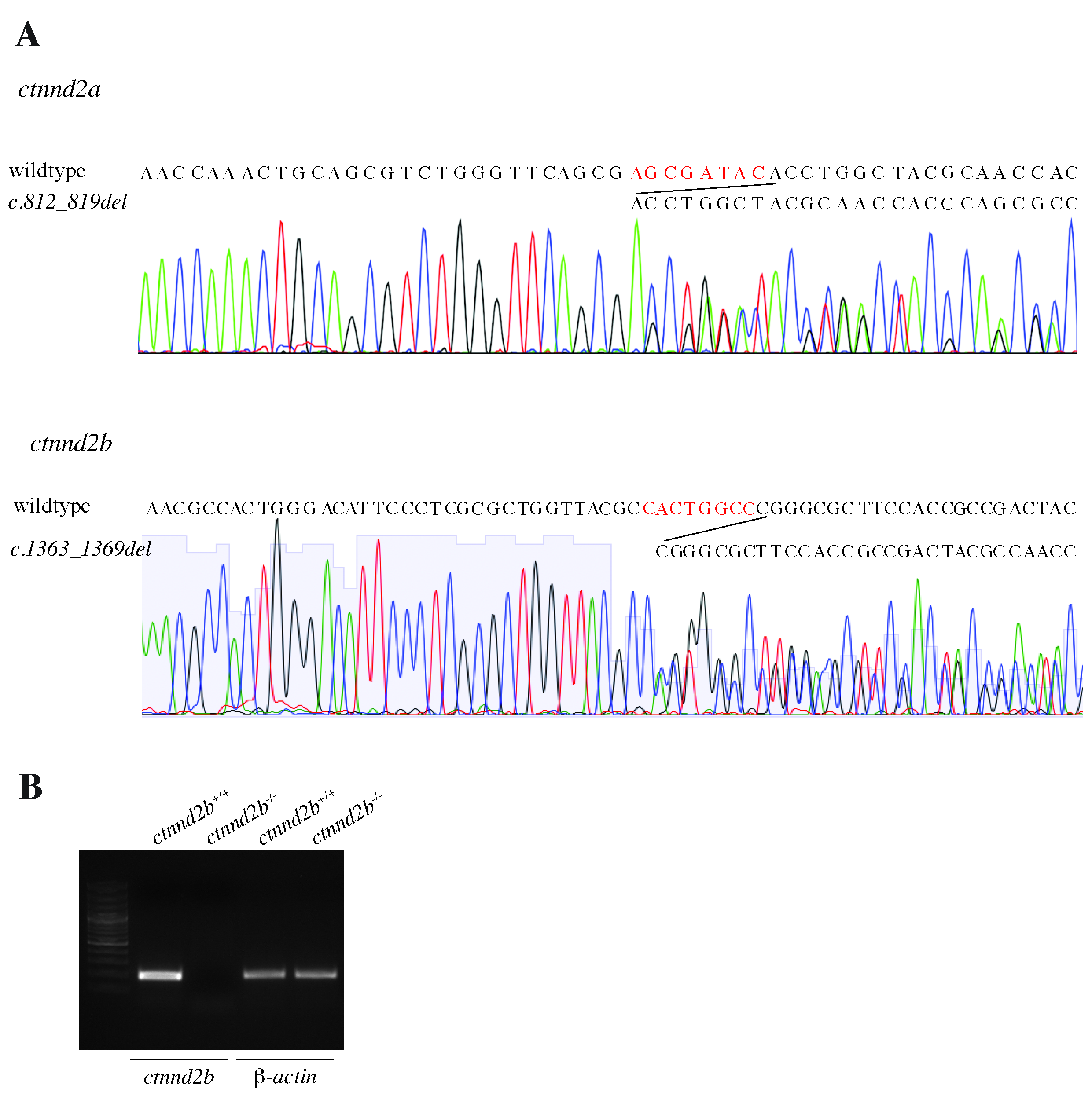

Supplement: Supplementary file 2 [file Image_1.TIFF]

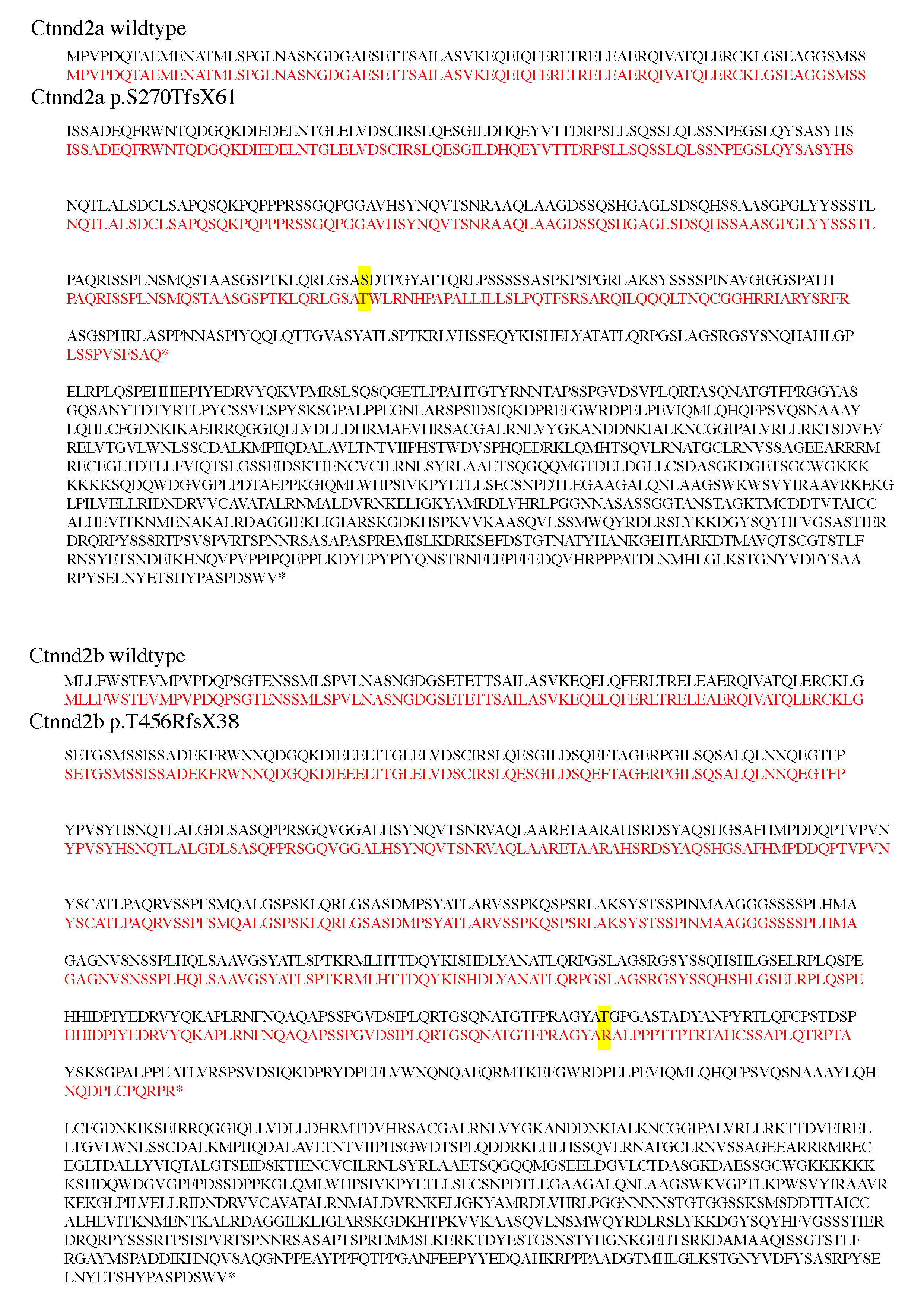

Supplement: Supplementary file 3 [file Image_2.TIFF]

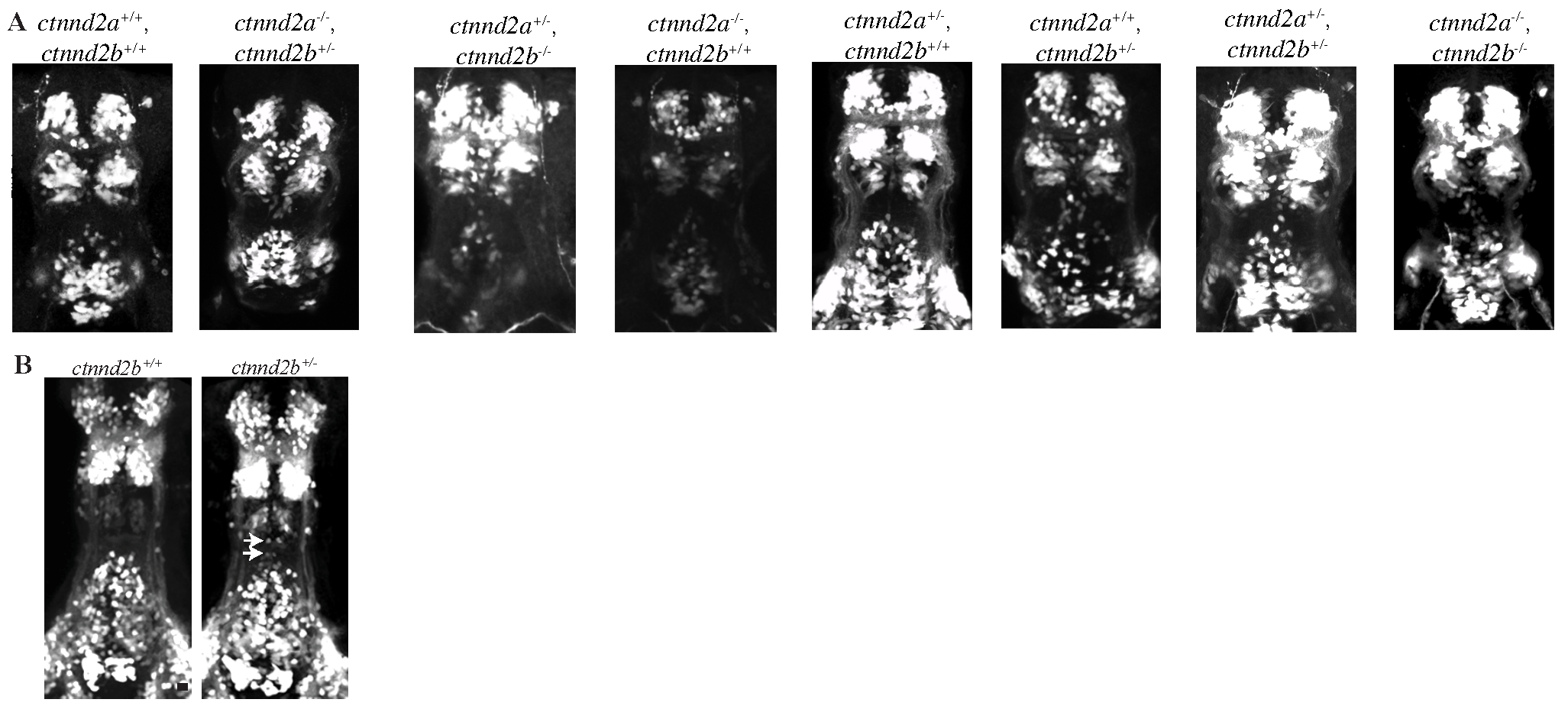

Supplement: Supplementary file 4 [file Image_3.TIFF]
